# Stability of context in sport and exercise across educational transitions in adolescence: hello work, goodbye sport club?

**DOI:** 10.1186/s12889-021-12471-4

**Published:** 2022-01-21

**Authors:** Vanessa Gut, Julia Schmid, Lars Imbach, Achim Conzelmann

**Affiliations:** Institute of Sport Science, University of Bern, Bremgartenstrasse 145, 3012 Bern, CH Switzerland

**Keywords:** Physical activity, Youth, Person-oriented approach, Social-ecological framework, Life event

## Abstract

**Background:**

The present study firstly aimed to identify context patterns in sport and exercise among adolescents at lower and upper secondary education. The organisational, social and competitive contexts of leisure-time sport and exercise were included as pattern indicators. The second aim was to examine the stability of these patterns across educational transition. The last aim was to investigate whether a subjective evaluation of the transition influences whether people stay in the same pattern across time.

**Methods:**

One-year longitudinal data of 392 adolescents were analysed.

**Results:**

Both before and after the educational transition, four context patterns were identified: the traditional competitive club athletes with friends, the self-organised individualists, the non-club-organised sportspersons and the mostly inactives. More than half of the individuals stayed in the same pattern across time. When individuals changed pattern, their change was most often from the self-organised individualists and the non-club-organised to the mostly inactives. A subjective evaluation of the transition influenced the stability of only the traditional competitive club athletes with friends. The chance of these people staying in the same pattern decreased with increased transitional stress.

**Conclusions:**

Knowledge about the stability and change of context patterns can be used to make recommendations for policy strategies and to develop more individually-tailored promotion programs.

**Supplementary Information:**

The online version contains supplementary material available at 10.1186/s12889-021-12471-4.

## Introduction

The transition from lower to upper secondary education^1^, occurring between 14 and 16 years of age, can be a major life event for many adolescents. They are faced with different challenges, such as a changed environment, new social roles or higher academic demands [2]. Indeed, there is evidence that this educational transition influences the sport and exercise behaviour of these young people [3]. For instance, a nationally representative longitudinal study from the Netherlands revealed that sport and exercise frequency decreased by around 20% when an individual left compulsary school [4]. Considering the various health benefits of physical activity [5, 6], it is vital to promote it among adolescents during their educational transition. However, to develop policy strategies and interventions, it is essential not only to identify factors that influence physical activity behaviour, but also to understand their interplay within each individual. In the present study, we concentrate on sport and exercise, two similar subsets of physical activity. Both are planned, structured, and performed during leisure time [7].

According to the social-ecological framework [8], an individual’s activity levels are impacted by behavioural context. Hence, this paper focuses on the 1) organisational, 2) social and 3) competitive context of sport and exercise. The *organisational context* refers to the setting in which adolescents do their sport and exercise. For example, they can participate in club activities (hereinafter referred to as a club-organised context), which are typically characterised by regular training sessions, coupled with the expectation that club members will voluntarily help out with additional club activities [9]. Alternatively, adolescents can be physically active with a commercial provider (e.g., gym, dance studio; hereinafter referred to as a non-club-organised context). Such activities have a similar organisational structure, but fewer social obligations [9]. Finally, adolescents may take part in self-organised activities that are flexible in terms of time (e.g., in informal groups; hereinafter referred to as a self-organised setting). The *social context* refers to the people with whom adolescents do their sport and exercise, i.e. with their family, friends, strangers, or alone. The *competitive context* refers to the mode of an activity. Adolescents can engage in competitive environments, where two or more rivals vie against one another, or they can opt for more recreational exercises.

The social-ecological framework [8] is supported by empirical research which shows that the aforementioned contextual factors are associated with maintaining exercise and sport. Thus, doing sport and exercise in a club during the years of adolescence is positively linked with being persistently active later on in life [10–13]. In contrast, doing organised, non-club activities (e.g., gym, using facilities offered by municipalities and private companies) has been associated with dropout [9, 14]. Furthermore, adolescents who do sport and exercise with peers are more likely to lead an active lifestyle in adulthood [15] because the underlying mechanisms, such as emotional and instrumental support or motivation that serve to promote adolescents’ sport and exercise behaviour [16] are present. Finally, competitive sport activities in adolescence are seen to make a difference in maintaining sport and exercise [17]. Studies have also indicated sex differences for all of the aforementioned behavioural contexts. For example, boys are more likely to engage in club-organised, competitive activities than girls (e.g., [18]).

Since, the context factors are related to sport and exercise behaviour, the question arises whether these factors change during adolescence and young adulthood. There is empirical evidence that exercise and doing sport in clubs decreases with age [9, 10, 19]. Furthermore, competitive sports activities in a team or as an individual decline when entering in adulthood [19, 20]. This observed move away from club-based, competitive activities towards a more self-organised setting may be explained by increased educational demands and thus an increased need for flexibility [21].

Although there is some knowledge about change in the context of sport and exercise during adolescence and young adulthood, further empirical research is indicated. An important issue for future research is the focus on typical context patterns in sport and exercise. Up to now, behavioural contexts have been studied mostly in isolation (e.g., [17]), ignoring the fact that they do, indeed, interact. For instance, adolescents are more likely to participate in competitions if they are in a club setting (e.g., [20]). Therefore, it is relevant to examine not only which behavioural context patterns exist, but also how stable they are over time. Person-oriented studies focus on such configurations, which show how contexts interact *within* an individual [22, 23]. The majority of these studies, have a relatively narrow focus because their investigation of patterns is limited to an organisational context of sport and exercise and/or in activity types (e. g., [24–26]). In this research, however, five to six different patterns were often identified. On the one hand, there were the traditional patterns, characterised either by club-organised team activities or as activities undertaken by an individual athlete. In contrast, more recreational patterns revealed activities that were less formal in nature and not club-organised (e.g., running, fitness). A recent cross-sectional study by Gut et al. [27] took a broader perspective and identified the following four patterns in adolescents: mostly inactives, non-club-organised individualists, self-organised individualists and family sportspersons, and the traditional competitive club athletes with friends. However, it was not investigated how stable these patterns were over time. In the person-oriented approach, researchers are usually interested in the effect of stability, on both group and individual levels. On a group level, so-called structural stability is high if patterns before and after the educational transition are replicated in similar forms (i.e. indicator means of the behavioural context patterns are more or less the same). Individual stability, on the other hand, refers to course of development between behavioural context patterns. If the majority of individuals are shown to belong to the same pattern both before and after the transition, individual stability is given [28].

Prospective longitudinal studies focusing on the transition from lower to upper secondary education are an additional issue for further research. Although there are several studies with a life-course perspective (e.g., 9), less is known about how the context of sport and exercise is impacted by this particular educational transition. Of interest is not only how the objective characteristic of this life event influences the context of sport and exercise, but also the adolescents’ subjective evaluation of the event. It is likely that the transition is not perceived in the same way by all individuals and this, in turn, may affect behaviour [29, 30]. We can assume that individuals who evaluate the transition as stressful may be more prone to changing the context of their sport and exercise. In fact, existing research shows that when stress is high, people are more likely to engage in non-competitive sport and exercise activities, for example [31].

In light of these two research gaps, the present study examined the following research questions:

1. Which behavioural context patterns in sport and exercise among adolescents exist before and after transitioning from lower to upper secondary education?

We identified patterns from three organisational context factors (club-organised, non-club-organised, self-organised settings), participation in competitions, as well as four social context factors (doing sport and exercise with friends, family/partner, people one does not know, or alone). Due to the relatively sparse person-oriented research in this area [13, 27], we did not expect a specific number of patterns. However, we hypothesised the existence of a “traditional” pattern, including club-organised competitive activities with friends [25, 27]. We then compared the detected patterns in terms of current sport and exercise behaviour, gender, and type of sport and exercise. In line with existing research, we expected individuals in “traditional” patterns to have a higher sport and exercise volume and to be more often boys [25]. In contrast, we had no *a priori* hypothesis regarding differences in type of sport and exercise.

2. How stable are the behavioural context patterns in sport and exercise across educational transition?

In this study, we examined stability firstly at a group level by focusing on the structural stability of context patterns. Secondly, we examined it at an individual level, by analysing how many people stay in the same pattern and how many people change patterns across educational transition. Since there is no longitudinal research on the stability of behavioural context patterns, we pursued an explorative approach with no prior assumption on this topic.

3. Are the associations between behavioural context patterns before and after transition moderated by the subjective evaluation of educational transition?

Based on theoretical considerations [30] and initial empirical evidence [13, 31], we expected the subjective evaluation of the life event to influence stability in behavioural context patterns. More specifically, we assumed that the higher the transitional stress, the more likely a change in the behavioural context pattern would occur.

Addressing these questions helps to effectively promote sport and exercise. If academics, practitioners and policymakers are informed about the behavioural context patterns and their development with age, they can use this knowledge to make recommendations for promotion strategies and to develop more individually-tailored programs.

## Methods

### Participant recruitment and procedures

In this longitudinal study, adolescents were recruited from 77 different school classes in Switzerland. The first data collection took place in spring 2016 (*t*_1_), when adolescents were in their 9^th^ and last year of the lower secondary school. The second data collection was conducted one year later in spring 2017 (*t*_2_), after these adolescents had switched to either vocational education and training (VET), a baccalaureate school or a transitional option. At *t*_1_, adolescents filled out a paper-pencil questionnaire during school lessons under the supervision of one of the authors. Adolescents were asked to provide their postal and mail-contact information for a second survey. At *t*_2_, adolescents were contacted with a link to an online questionnaire. Participants who filled out both surveys received a voucher for 15 CHF. From the 953 adolescents at *t*_1_, 392 participated a second time. Students were excluded from the study if they had psychological or physical disabilities or were not proficient in German. Adolescents provided their informed written consent for the study. The ethics commission of the Faculty of Human Sciences of the University of Bern approved the study design and procedures.

### Participant characteristics and study dropout

Characteristics of the sample are summarised in the Electronical Supplementary Material, see Table S1. Study dropout analyses were conducted to compare those who were eligible for the study but did not participate at *t*_2_ (dropouts, *n* = 553, 58.5%) with those who completed both questionnaire assessments (completers, *n* = 392, 41.5%) in terms of context and socio-demographic variables. No differences were found for sport and exercise activity level (minutes per week) and all behavioural context variables. However, t-tests and Chi-square tests showed that the study dropout rate was lower for the older participants (*t*(865.926) = -2.618, *p*_bonferroni-corrected_ = .045, *d* = 0.089; 95% CI [0.027, 0.186]), girls (χ^2^ (1) = 15.541, *p* < .0005, *ϕ*_*corr*_ = 0.281, 95% CI [.225, .353]), Swiss (χ^2^ (1) = 8.488, *p* = .004, *ϕ*_*corr*_ = 0.231, 95% CI [.171, .299]) and those with a B school-level (χ^2^ (1) = 24.121, *p* < .0005, *ϕ*_*corr*_ = 0.317, 95% CI [.264, .392]). As the reported effect sizes are rather small, the sample might be biased negligibly.

### Measures

#### Volume and type of sport and exercise

Sport and exercise volume was measured at *t*_1_ and *t*_2_ with the BSA questionnaire [32]. Participants named a maximum of three sport and exercise activities they had regularly engaged in within the last four weeks. They indicated the frequency and duration per episode in minutes for each activity. Based on this information, an overall index value in “min per week” was calculated. In addition, the weekly time in different activity types was computed and transformed into percentage variables (e.g., of the 100 minutes of sport and exercise per week, 60 minutes were football and 40 minutes jogging, resulting in a percentage value of 60% for the first activity type and 40% for the second activity).

#### Context of sport and exercise

Right after the BSA questionnaire, adolescents were asked to choose their organisational context: Did they engage in the previously named sport and exercise activities (a) in a club, (b) a commercial provider, or (c) was it self-organised? They were also asked in which social context: Were they (a) alone, (b) with friends, (c) with family/partner or (d) with strangers? Furthermore, for each of the activities, the adolescents were asked if they participated in competitions. Afterwards, the weekly time in these different contexts was calculated (e.g., of the 100 min of sport and exercise per week, 60 min were done at the club and 40 minutes were self-organised) and converted into percentage variables (e.g., 60% were done at the club and 40% were self-organised).

#### Subjective evaluation of the educational transition

The subjective evaluation of the educational transition was assessed at *t*_2_ with a five-item inventory for life-changing events [33, 34]. Participants rated four aspects of the transition on a 5-point-likert-scale ranging from 1 (*not true*) to 5 (*totally true*): uncontrollability (e.g., “at first I was completely at the mercy of the event”), unpredictability (e.g., “the event was unpredictable for me”), impact (e.g., “the event forced me to plan my everyday life differently”) and centrality (e.g., “the event hit me at my very core”). The internal consistency of the inventory was good (α = 0.75).

### Statistical analyses

To identify context patterns in sport and exercise among adolescents in lower and upper secondary school (Research question [RQ] 1), latent transition analysis was conducted [35]. All analyses were run in Mplus Version 8.0 [36] using maximum likelihood estimation with robust standard errors (MLR). For six adolescents it was not possible to calculate the targeted percentage value (0.31% missing data). The missing data were accommodated via full-information maximum likelihood (FIML). In the first step, two to six context patterns were separately calculated for each measurement time using eight context factors. To decide the optimal number of context patterns, statistical and content-related criteria were considered. As statistical criteria, the Bayesian information criterion (BIC), sample-adjusted BIC, and the Bootstrapped likelihood-ratio test (BLRT) were used. The elbow-criterion was applied for BIC and sample-adjusted BIC [37]. In addition, content-related criteria, such as the principle of parsimony, conformity with theoretical considerations and interpretability were applied. Z-Scores were utilised to better interpret and label the patterns. To further validate the identified patterns, sport and exercise activity level, gender distribution, and type of sport and exercise activity were described and differences in these factors investigated with Wald-tests [38]. Structural stability of the patterns (RQ 2a) was examined with a measurement invariance test [37]. To investigate individual stability (RQ 2b), transition probabilities were calculated. To examine the overall moderation effect of a subjective evaluation of the transition on associations between the context patterns (RQ 3), a likelihood-ratio chi-square-test was conducted [39]. A model with free slopes for the subjective evaluation (interaction model) was compared with a model with slopes fixed at 0 (no interaction model). Additionally, a multinominal logistic regression analysis was performed on a latent level. This follow-up analysis allows interaction effects to be investigated in more detail (e.g., which specific pattern has an interaction effect and which does not).

## Results

### Behavioural context patterns in sport and exercise

For both measurement points, two- to eight-pattern-solutions were tested. In general, BIC and sample-adjusted BIC improved with more patterns (see Electronical Supplementary Material, Table S2). BLRTs were significant for all pattern solutions. The elbow-criterion pointed to a three- to five-pattern model. However, in the five-pattern solutions, convergence problems occurred when conducting latent transition analysis. A closer inspection of the content-related indicators revealed the four-pattern solutions as the most favoured model for both *t*_1_ and *t*_2_ (see Tables 1 and 2)_._ In the three-pattern solutions, patterns varied only in organisational context factors and thus were considered to fail to adequately differentiate. The four-pattern solution for *t*_1_ and *t*_2_ consists of the following behavioural context patterns: (1) *traditional competitive club athletes with friends*, (2) *self-organised individualists*, (3) *non-club-organised sportspersons* and (3) *mostly inactives*.

**Table 1:** Context patterns before educational transition (t_1_): latent profile analysis models for 3- to 5-latent-pattern solutions (class-invariant, diagonal Σ)

Latent-pattern solution	*n* (%)	Club-organised *M (SD)*	Non-club organised *M (SD)*	Self-organised *M (SD)*	Alone *M (SD)*	With people you do not know *M (SD)*	With family and/or partner *M (SD)*	With friends *M (SD)*	Competition participation *M (SD)*	Sport and exercise volume *M (SD)*	Sex	BIC	Sample-adjusted BIC	Entropy	VLMR	BLRT^a^
*Three latent patterns*												429.19	321.305	.98	*p* < .00005	-397.954 *p* < .00005
1. Self-organised individualists	164 (41.84%)	3.90% (1.41)	2.00% (1.05)	45.20% (3.10)	22.00% (2.86)	5.80% (2.36)	6.40% (1.64)	27.00% (3.62)	7.70% (1.01)	152.00 min/week (21.08) [2, 3]	67.50% [2, 3]					
2. Non-club-organised sportspersons	50 (12.76%)	4.10% (1.41)	84.70% (1.05)	8.60% (3.10)	26.50% (2.86)	12.00% (2.36)	7.70% (1.64)	40.90% (3.62)	12.50% (1.01)	250.33 min/week (30.08) [1, 3]	82.00% [1, 3]					
3. Traditional competitive club athletes with friends	178 (45.41%)	87.90% (1.41)	3.10% (1.05)	7.10% (3.10)	7.10% (2.86)	9.30% (2.36)	3.50% (1.64)	76.90% (3.62)	68.00% (1.01)	319.67 min/week (17.00) [1, 2]	51.70% [1, 2]					
									Wald-Chi-Square-Test	χ^2^ = 37.56, *p* < .00005	χ^2^ = 22.30, *p* < .00005					
*Four latent patterns*												-52.02	-188.457	.98	*p* < .00005	-113.081 *p* < .00005
1. Traditional competitive club athletes with friends	179 (45.66%)	87.70% (1.38)	3.20% (1.05)	7.30% (1.34)	7.20% (1.34)	9.60% (25.69)	3.70% (23.66)	76.40% (35.78)	67.40% (32.09)	318.98 min/week (16.86) [2, 3,]	51.90% [2, 3]					
2. Mostly inactives	81 (20.66%)	1.30% (1.38)	1.30% (1.05)	2.50% (1.34)	2.80% (1.34)	5.40% (25.69)	3.50% (23.66)	17.20% (35.78)	7.50% (32.09)	56.51 min/week (15.74) [1, 3, 4]	70.70% [1]					
3. Non-club-organised sportspersons	51 (13.01%)	4.40% (1.38)	84.20% (1.05)	8.80% (1.34)	26.50% (1.34)	12.80% (25.69)	7.80% (23.66)	41.00% (35.78)	12.70% (32.09)	247.01 min/week (29.76) [1, 2]	82.20% [1, 4]					
4. Self-organised individualists	81 (20.66%)	5.50% (1.38)	2.30% (1.05)	89.40% (1.34)	41.80% (1.34)	5.10% (25.69)	9.10% (23.66)	36.90% (35.78)	7.60% (32.09)	247.90 min/week (36.63) [2]	63.80% [3]					
									Wald-Chi-Square-Test	χ ^2^ = 135.21, *p* < .00005	χ ^2^ = 23.204, *p* < .00005					
*Five latent patterns*												-388.152	-553.146	.99	*p* = .0398	154.392 *p* < .00005
1. Mostly inactives	79 (20.15%)	0.70% (1.41)	0.30% (0.55)	2.10% (1.34)	2.50% (2.57)	3.60% (2.35)	3.10% (1.64)	16.80% (3.56)	6.00% (3.19)	46.64 min/week (13.96) [2, 3, 4, 5]	71.00% [3]					
2. Non-club-organised sportspersons	35 (8.93%)	0.00% (1.41)	96.00% (0.55)	2.50% (1.34)	22.90% (2.57)	14.00% (2.35)	10.10% (1.64)	38.90% (3.56)	10.90% (3.19)	235.94 min/week (38.03) [1, 3, 4, 5]	86.30% [3, 4, 5]					
3. Traditional competitive club athletes with friends	167 (42.60%)	89.80% (1.41)	0.90% (0.55)	7.50% (1.34)	6.40% (2.57)	9.30% (2.35)	3.8% (1.64)	77.20% (3.56)	69.10% (3.19)	306.04 min/week (16.86) [1, 2, 5]	53.30% [1, 2]					
4. Self-organised individualists	78 (19.90%)	6.00% (1.41)	0.70% (0.55)	90.50% (1.34)	42.00% (2.57)	5.30% (2.35)	9.50% (1.64)	35.70% (3.56)	8.10% (3.19)	252.70 min/week (37.93) [1, 2]	62.60% [2]					
5. Flexible sportspersons	33 (8.42%)	28.50% (1.41)	47.60% (0.55)	19.10% (1.34)	27.20% (2.57)	14.40% (2.35)	2.90% (1.64)	53.80% (3.56)	27.30% (3.19)	348.43 min/week (40.78) [1, 2, 3]	59.80% [2]					
									Wald-Chi-Square-Test	χ^2^ = 168.74, *p* < .00005	χ^2^ = 21.298, *p* < .00005					

*Note.* BIC = Bayesian information criterion; sample-adjusted BIC = sample-adjusted Bayesian information criterion; VLMR = Vuong-Lo-Mendell-Rubin likelihood ratio test; BLRT = bootstrapped likelihood-ratio test. Numbers in brackets indicate that patterns differ significantly at *p* < .05. ^a^The BLRT could only be performed without correction for nesting.

**Table 2:** Behavioural context patterns after the educational transition (t_2_): latent profile analysis models for 3-to 5-latent-pattern solutions (class-invariant, diagonal Σ)

Latent-pattern solution	*n* (%)	Club-organised *M (SD)*	Non-club organised *M (SD)*	Self-organised *M (SD)*	Alone *M (SD)*	With people you do not know *M (SD)*	With family and/or partner *M (SD)*	With friends *M (SD)*	Competition participation *M (SD)*	Sport and exercise volume *M (SE)*	Sex *M (SE)*	BIC	Sample-adjusted BIC	Entropy	VLMR	BLRT^a^
*Three latent patterns*												-145.069	-252.950	.99	*p* = .0014	-220.888 *p* < .00005
1. Mostly inactives	189 (48.32%)	1.40% (1.22)	0.90% (0.89)	33.10% (3.27)	20.90% (3.11)	1.60% (1.73)	3.40% (1.70)	14.20% (3.13)	2.70% (2.68)	92.71 min/week (12.73) [2, 3]	64.00% female					
2. Traditional competitive club athletes with friends	152 (38.78%)	86.50% (1.22)	3.10% (0.89)	8.80% (3.27)	0.90% (3.11)	8.60% (1.73)	1.90% (1.70)	79.70% (3.13)	66.00% (2.68)	354.36 min/week (22.04) [1, 3]	57.70% female					
3. Non-club-organised sportspersons	51 (13.01%)	0.30% (1.22)	89.90% (0.89)	6.40% (3.27)	28.00% (3.11)	11.00% (1.73)	18.10% (1.70)	41.90% (3.13)	8.70% (2.68)	278.88 min/week (27.86) [1, 2]	69.40% female					
									Wald-Chi-Square-Test	χ^2^ = 121.62, *p* < .00005	χ^2^ = 2.72, *p* = .26					
*Four latent patterns*												-868.684	-1005.122	1.00	*p* < .00005	174.046 *p* < .00005
1. Non-club-organised sports persons	51 (13.01%)	3.00% (1.23)	90.40% (0.89)	5.90% (1.18)	27.40% (2.49)	11.10% (1.73)	18.30% (1.70)	42.20% (3.00)	8.70% (2.68)	278.73 min/week (27.91) [2, 3]	69.20% female					
2. Mostly inactives	121 (30.87%)	0.00% (1.23)	0.00% (0.89)	0.01% (1.18)	0.01% (2.49)	0.00% (1.73)	0.01% (1.70)	4.70% (3.00)	0.10% (2.68)	23.33 min/week (10.99) [1, 3, 4]	66.20% female					
3. Traditional competitive club athletes with friends	152 (38.78%)	86.50% (1.23)	3.10% (0.89)	8.70% (1.18)	8.90% (2.49)	8.60% (1.73)	1.90% (1.70)	79.80% (3.00)	66.10% (2.68)	354.39 min/week (22.08) [1, 3, 4]	57.60% female					
4. Self-organised individualists	68 (17.35%)	4.10% (1.23)	2.80% (0.89)	91.50% (1.18)	56.80% (2.49)	3.70% (1.73)	7.90% (1.70)	31.50% (3.00)	6.40% (2.68)	218.00 min/week (22.44) [2, 3]	60.30% female					
									Wald-Chi-Square-Test	χ ^2^ = 241.75, *p* < .00005	χ^2^ = 3.40, *p* = .334					
*Five latent patterns*												-1285.32	-1450.309	1.00	*p* = .0398	562.724 *p* < .00005
1. Mostly inactives	121 (30.87%)	0.00% (1.05)	0.00% (0.89)	0.40% (0.71)	0.90% (2.49)	0.40% (1.73)	0.80% (1.70)	4.50% (3.00)	0.80% (2.61)	23.33 min/week (10.99) [2, 3, 4, 5]	66.10% female					
2. Non-club-organised sportspersons	30 (7.65%)	42.70% (1.05)	7.80% (0.89)	46.70% (0.71)	28.40% (2.49)	7.10% (1.73)	2.90% (1.70)	61.40% (3.00)	35.10% (2.61)	324.25 min/week (48.88) [1, 5]	50.20% female					
3. Self-organised individualists	51 (13.01%)	3.20% (1.05)	90.30% (0.89)	5.80% (0.71)	27.30% (2.49)	11.10% (1.73)	18.20% (1.70)	42.50% (3.00)	8.90% (2.61)	279.04 min/week (27.80) [1, 4]	68.80% female					
4. Traditional competitive club athletes with friends	133 (33.93%)	90.90% (1.05)	3.10% (0.89)	4.30% (0.71)	7.50% (2.49)	9.10% (1.73)	1.80% (1.70)	80.90% (3.00)	69.50% (2.61)	353.39 min/week (23.20) [1, 3, 5]	60.00% female					
5. Flexible sportspersons	57 (14.54%)	0.50% (1.05)	0.30% (0.89)	98.40% (0.71)	59.50% (2.49)	2.70% (1.73)	8.90% (1.70)	28.90% (3.00)	2.50% (2.61)	207.68 min/week (24.21) [1, 4]	59.60% female					
									Wald-Chi-Square-Test	χ^2^ = 242.18, *p* < .00005	χ^2^ = 3.87, *p* = .424					

*Note.* BIC = Bayesian information criterion; sample-adjusted BIC = sample-adjusted Bayesian information criterion; VLMR = Vuong-Lo-Mendell-Rubin likelihood ratio test; BLRT = bootstrapped likelihood-ratio test. Numbers in brackets indicate that patterns differ significantly at *p* < .05.

The traditional competitive club athletes with friends make up the largest pattern (*t*_1_: *n* = 179; 45.66%; *t*_2_: *n* = 152, 38.78%). They are characterised by doing sport and exercise with friends in a club and participating in competitions. Compared to the overall sample, there is an above average number of young males in this pattern (*t*_1_: 49.10%, *t*_2_: 42.40%). The traditional competitive club athletes with friends are the most active (*t*_1_: 318.98 min sport and exercise/week, *t*_2_: 297 min sport and exercise/week). They practise primarily sports games (*t*_1_: 68.50%, *t*_2_: 66.90%; see Electronical Supplementary Material, Table S3). The self-organised individualists (*t*_1_: *n* = 81, 20.66%; *t*_2_: 68, 17.35%) often do sport and exercise in a self-organised context and are mainly active alone, compared to the other adolescents. 63.80% (*t*_1_) and 60.30% (*t*_2_) respectively were females. Adolescents in this pattern are relatively active (*t*_1_: 247.90 min, *t*_2_: 218.00 min sport and exercise/week). While many carry out fitness (23.60%) or sports games (27.80%) at *t*_1_, the percentage values shift slightly at *t*_2_ (fitness: 32.30% vs. sports games: 18.50%). The non-club-organised sportspersons (*t*_1_ and *t*_2_: *n* = 51; 13.01%) do their sport and exercise mainly in a non-club-organised setting, such as a gym or a dance studio. This pattern has the highest percentage of females (*t*_1_: 82.20%, *t*_2_: 69.20%). The non-club-organised sportspersons are relatively active, with 247.01 min (*t*_1_) and 278.73 min (*t*_2_) of sport and exercise per week. At *t*_1_, they practise compositional-creative activities mainly, such as dancing (44.40%) and fitness (22.20%). At *t*_2_, the weekly volume of fitness activities increases (46.90%), while that of compositional-creative activities decreases (26.50%). The mostly inactives (*t*_1_: *n* = 81, 20.66%; *t*_2_: 121, 30.87%) do no sport and exercise or only a little (*t*_1_: 51.51 min, *t*_2_: 23.33 min sport and exercise/week). Consequently, they have average to under-average z-values in all context factors. In this pattern, 70.70% (*t*_1_) and 66.20% (*t*_2_) are females. If they are active, they practise mainly sports games (24.80%) and relaxation-oriented activities, which lower energetic arousal, such as yoga or tai chi (21.20%) at *t*_1._ Only the proportion of the sports games remains stable at 47.10% at *t*_2_.

### Structural and individual stability of the context patterns across educational transition

**Fig. 1:**
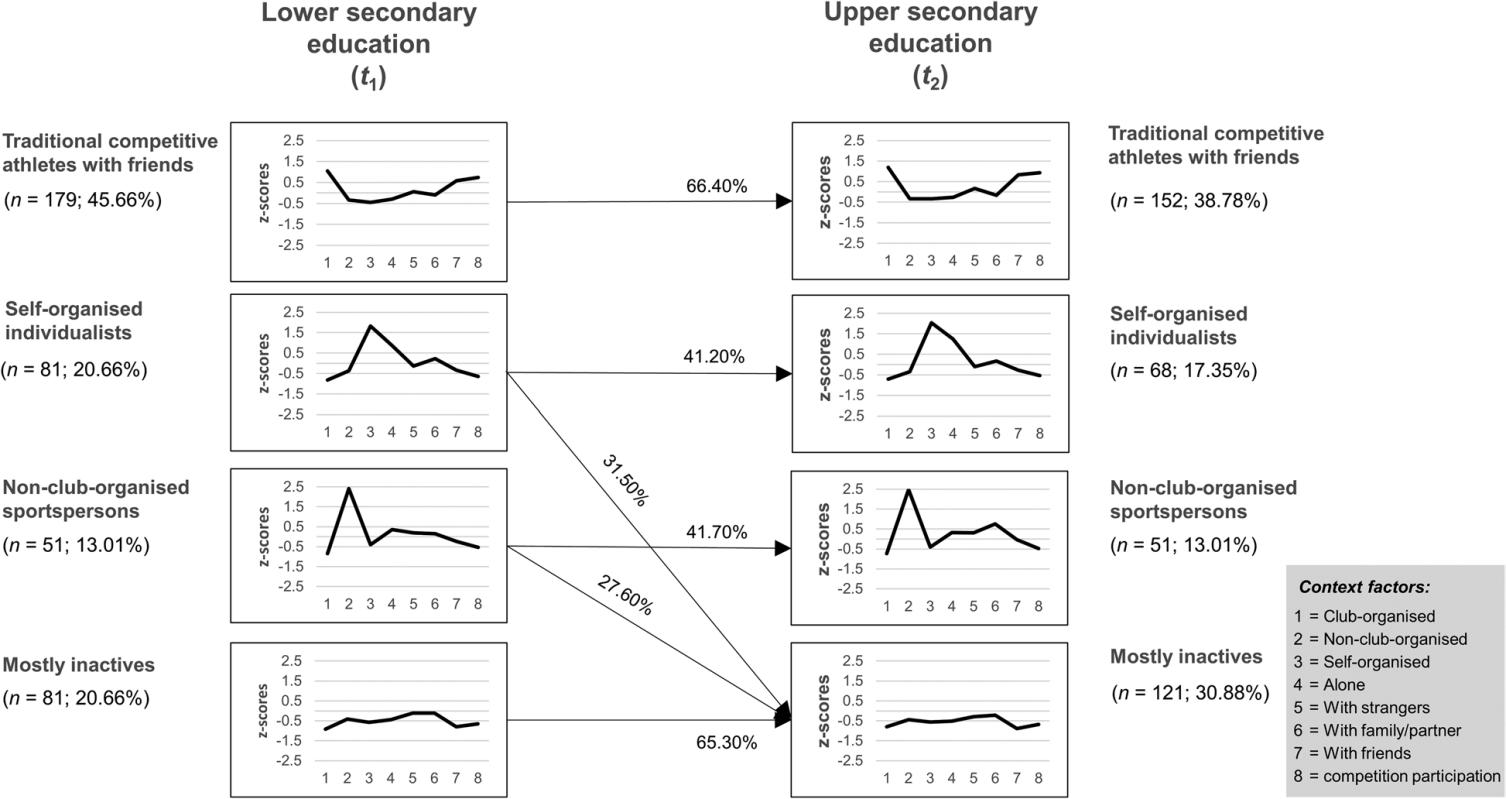
Z-standardised behavioural context patterns (t1 and t2) their transitional probabilites (arrows). Only transition probabilities ≥ 20% are reported

The structural measurement invariance testing (χ^2^ = 29.90, *p* = .57) indicates that patterns are stable over time and can be interpreted in the same way before and after educational transition. Overall, 58.20% (*n* = 228) of all adolescents stay in the same context pattern over time. However, the transition probabilities in Fig. 1 show that the percentages vary between patterns. The traditional competitive club athletes with friends (66.40%) and the mostly inactives (65.30%) most often remain in the same pattern. In contrast, the proportion of remainers is smaller in the self-organised individualists (41.20%) and the non-club-organised sportspersons (41.20%). Some people in these two patterns switch to the mostly inactives after educational transition (31.50% and 27.60%, respectively).

### The moderating effect of a subjective evaluation of the transition on stability in behavioural context patterns

The likelihood-ratio chi-square-test revealed an overall moderation effect of a subjective evaluation (χ^2^[*df* **= **15]** =** 23.24, *p* **=** .0791). Table 3 reports the findings of the multinominal logistic regression analysis for each pattern, whereby the mostly inactives served as a reference group. Results showed that the competitive club athletes were less likely to be in the same pattern after the educational transition when they perceived the transition as stressful (*B* = -0.781 [CI 95%: -1.317, -0.246], *p* = .004).

**Table 3:** The moderating effects of a subjective evaluation of the educational transition on the associations of context patterns

	Upper secondary education (*t*_2_)							
	Traditional competitive club athletes with friends		Non-club-organised sportspersons		Self-organised individualists		Mostly inactives	
	*B* [95% CI]	*p*-value	*B* [95% CI]	*p*-value	*B* [95% CI]	*p*-value	*B* [95% CI]	*p*-value
Lower secondary education (*t*_1_)								
Traditional competitive club athletes with friends x subjective evaluation	-0.781 [-1.317, -0.246]	.004*	-0.402 [-1.052, 0.248]	.225	-0.50 [-1.187, 0.184]	.152	−	−
Non-club-organised sportspersons x subjective evaluation	0.243 [-0.280, 1.165]	.614	-0.453 [-0.325, 1.397]	.239	0.056 [-0.563, 0.841]	.886	−	−
Self-organised individualists x subjective evaluation	0.443 [-0.280, 1.165]	.230	0.536 [-0.325, 1.397]	.222	0.139 [-0.563, 0.841]	.698	−	−
Mostly inactives x subjective evaluation	-0.149 [-0.855, 0.556]	.678	-0.062 [-0.772, 0.648]	.864	0.304 [-0.486, 1.094]	.451	−	−

*Note.* For the analyses of the main effects of the psychosocial profiles, one-sided significance tests were applied. *B* = unstandardised B-regression coefficient; * *p* < .05. CI = confidence interval for unstandardised B-regression coefficient. Reference groups: Mostly inactives. For the moderation analysis, two-sided significance tests were applied.

## Discussion

In the present study, behavioural context patterns in exercise and sport were identified and their stability across educational transitions examined. Furthermore, it was tested whether associations between patterns over time are moderated by a subjective evaluation of educational transition.

Both before and after the educational transition, four behavioural context patterns were identified: (1) the traditional competitive club athletes with friends, (2) the self-organised individualists, (2) the non-club-organised sportspersons and (3) the mostly inactives. The patterns found are very similar to those identified by Gut et al. [27]. As hypothesised, the first pattern has a “traditional” character, as individuals doing sport and exercise with friends in a club and participating in competitions. The fact that this pattern consists of a relatively high number of males as well as active adolescents is also consistent with our hypothesis. An explanation for the high level of activity can be seen in the fact that competitive sport requires regular club training sessions [25]. It is not surprising that this pattern is the largest one, as previous variable-oriented [40] and person-oriented studies [27] show that the most popular context for adolescents is doing activities in clubs and with friends. The second and third patterns are smaller and characterised by more informal, flexible and non-competitive exercise contexts. The fact that the non-club-organised sportspersons have the highest number of females is in line with previous research, which highlights that female adolescents often favour commercial providers, such as a gym or a dance studio [19].

The identified behavioural context patterns are structurally stable across educational transition, meaning that they were very similar on a group level. Analysis on the individual level overall showed that around 60% of the adolescents stayed in the same behaviour context patterns, whereas around 40% moved to another pattern. However, a closer look at the results reveals that the likelihood of remaining in the same behaviour context pattern differs between patterns. The competitive club athletes with friends had the highest probability of remaining in the same highly active pattern, while that of the self-organised individualists and the non-club-organised sportspersons was much lower. Individuals of the latter two patterns often changed to the mostly inactives (see Fig. 1). To sum up, it seems that doing sport and exercise in a context with few social and organisational obligations during lower secondary education increases the risk of dropping out altogether from sport and exercise. In contrast, the sport clubs, with their regular training sessions and friends as a source of social support, may be factors beneficial for staying active across educational transition [12, 15].

As assumed, moderation analysis revealed overall that a subjective evaluation of the transition influenced the stability of behavioural context patterns across time. In more differentiated analyses, however, the moderation effect could be demonstrated for only one behaviour context pattern: the traditional competitive club athletes with friends. The chance of those people staying in the same pattern decreased with increased transitional stress. These findings illustrate that the objective characteristics of a life event and its subjective evaluation could have distinct impacts on stability and change of sport and exercise context [41, 42]. It is important to consider both aspects simultaneously as it provides a greater understanding of the mechanism [30]. However, the question arises as to why the traditional competitive club athletes with friends in particular are influenced by transitional stress. One of the characteristics of perceived high stress is that adolescents are forced to organise their everyday life differently or need to adapt their usual activities [33]. It can therefore be speculated that club training sessions with relatively fixed training times and places [9] were no longer compatible with the new life situation. Furthermore, competitive sport and exercise activities are often time-consuming and therefore difficult to maintain for adolescents with limited resources during stressful educational transitions [31]. In contrast, the self-organised individualists and the non-club-organised sportspersons may have already been strongly influenced by the objective characteristics of the life event and the perceived transitional stress did not have an additional effect on their sport and exercise context.

### Strengths, limitations and future directions

A strength of the present study is its focus on typical patterns. Whereas existing research has examined isolated context factors, the present research looked at the interplays of organisational, social and competitive factors *within* a person. Furthermore, this study adds value by its prospective longitudinal design and by the simultaneous consideration of an objective life event and the perception of transitional stress. The closer look at the transition from lower to upper secondary education seems to be particularly important because many adolescents decrease their amount of sport and exercise during this period [15]. However, we acknowledge some limitations. Firstly, the dropout analysis showed that the longitudinal study sample was distorted. Individuals were more likely to participate at both measurement points if they were older, girls, Swiss, and had reached B school-level. Such a self-selection runs the risk of rendering the sample no longer representative of the whole population of adolescents born between 2000 and 2001. However, one should keep in mind that the effect sizes were rather small and, therefore, may have affected our findings only marginally. Further, it is to be emphasised that the response rate (42%) was high compared to other longitudinal studies with youths [20]. Secondly, the diverse behaviour context factors were not included in the analyses in a balanced way (organisational context: four factors, social context: three factors, competitive context: one factor). Consequently, for instance, the organisational context had much more weight in the LTA, in relation to the competitive context [43]. It cannot be ruled out that this influenced the results of the present research. Thirdly, this study consists of only two measurement time points, one year apart. Although the longitudinal design already extends existing, mostly cross-sectional studies (e.g., [24, 27]), future studies should follow adolescents longer and interview them at multiple time points. With such a design, for example, it would be possible to study how a change in behaviour context pattern is associated with a change in sport and exercise volume or in health. Fourthly, the sample in the present study consisted of adolescents with diverse educational transitions (see Supplementary material, Table S1). The large majority of young people switched from lower secondary education to VET (61%). However, there were also individuals who changed from lower secondary education to baccalaureate school (28%) or transitional options (11%). It is possible that the type of transition experienced influenced the chance to stay in the same behaviour context pattern across time. Finally, to a certain extent, it remains unclear which factors boost change in behaviour context patterns and which protect against change. Although we were able to show that a subjective evaluation of the transition moderates individual stability [34], more in-depth studies using qualitative research methods would be interesting [44]. For instance, it can be assumed that psychological determinants play a protective role. Indeed, recent studies show that combining sport and exercise in a club with the individual’s ability to plan activities boosts the chances of staying active across educational transition [10–13]. Furthermore, it is conceivable that financial ressources influence individual stability of behaviour context patterns, because, for example, a sport club membership is typically less expensive in Switzerland than regular training in a gym. In line with the basic idea of the social-ecological framework [8], promotional measures should, therefore, be applied simultaneously at both the individual level (e.g., promotion of an individual’s volition) and the structural level (e.g., promotion of facility and access to sport clubs).

### Conclusion

The four behaviour context patterns found differ in their organisational, social and competitive settings of exercise and sport. More than half of the adolescents remained in the same pattern across their educational transition. Individuals, in particular, who did competitive sport with friends in a club during their lower secondary education are very likely to continue to do so. However, if these adolescents perceived high transitional stress, they often changed the context of sport and exercise and consequently said “goodbye” to their sport club.

The present study helps to better understand stability and changes in the context of sport and exercise behaviour, which is important for effectively promoting sport and exercise during the transition from lower to upper secondary education. Thus, the identified patterns can be used to develop policy strategies (e.g., promoting club-organised sport and exercise activities with regular training sessions and friends before educational transitions) and tailored interventions for specific subgroups of young people (e.g., empower the traditional competitive club athletes with friends to cope with transitional stress).

## Supplementary Information

**Additional file 1.**

**Additional file 2.**

**Additional file 3.**

## Data Availability

The datasets and syntaxes used during the current study are available from the corresponding author on request.

## Abbreviations

BIC: Bayesian information criterion
BLRT: Bootstrapped likelihood-ratio test
MLR: Maximum likelihood estimation with robust standard errors
RQ: Research question
VET: Vocational education and training

## Footnotes

1 The terms lower and upper secondary education refer to the international standard classification of education [1]. In many educational systems, the end of lower secondary education is the end of general, compulsory schooling. In contrast, upper secondary education typically prepares students more specifically for tertiary education or provides skills relevant for employment.
