# Supplementary material for: Stability of context in sport and exercise across educational transitions in adolescence: hello work, goodbye sport club?
Source: BMC Public Health. 2022 Jan 21;22:152. doi: 10.1186/s12889-021-12471-4 (PMC8783455; doi:10.1186/s12889-021-12471-4)
Supplement: Supplementary file 1 — Additional file 1. [file 12889_2021_12471_MOESM1_ESM.docx]

Table S1

*Sample characteristics*

| Characteristics | | Full sample *t*_1_  (*n* = 945) | Longitudinal sample *t*_1_*-t*_2_  (*n* = 392) |
| --- | --- | --- | --- |
| Sex | Female | 515 (54.5%) | 244 (62.2%) |
|  | Male | 427 (45.2%) | 148 (37.8%) |
|  | Not available | 3 (0.3%) | − |
| Age in lower secondary education (*t*_1_) | | *M* = 15.34, *SD* = 0.62 | *M* = 15.27, *SD* = 0.60 |
| Nationality | Swiss | 724 (76.6%) | 322 (82.1%) |
|  | Other | 176 (18.6%) | 57 (14.5%) |
|  | Not available | 45 (4.8%) | 13 (3.3%) |
| Level lower secondary education (*t*_1_) | level C^1^ | 248 (26.3%) | 70 (17.9%) |
|  | level B^2^ | 662 (70.2%) | 309 (78.8%) |
|  | Not available | 33 (3.5%) | 13 (3.3%) |
| Level upper secondary education (*t*_2_) | Vocational education and training (VET) | − | 240 (61.2%) |
|  | Bacccalaureate school | − | 111 (27.7%) |
|  | Transitional options | − | 41 (10.5%) |
| Exercise and sport in lower secondary education (*t*_1_) | No exercise and sport | 169 (17.9%) | 59 (15.1%) |
|  | 1-74 min/week | 106 (11.2%) | 47 (12.0%) |
|  | >74 min/week | 662 (70.1%) | 286 (73.0%) |
|  | Not available | 8 (0.8%) | − |

*Note.* ^1^Lower secondary school, level C includes “Realschule” and “Oberschule”; ^2^ Lower secondary school, level B includes “Bezirksschule” and “Sekundarschule”.
