# Supplementary material for: Stability of context in sport and exercise across educational transitions in adolescence: hello work, goodbye sport club?
Source: BMC Public Health. 2022 Jan 21;22:152. doi: 10.1186/s12889-021-12471-4 (PMC8783455; doi:10.1186/s12889-021-12471-4)
Supplement: Supplementary file 2 — Additional file 2. [file 12889_2021_12471_MOESM2_ESM.docx]

Table S2

| *t*_1_: Context patterns before educational transition | df | LL | BIC | Sample-adjusted BIC | Entropy | VLMR | BLRT |
| --- | --- | --- | --- | --- | --- | --- | --- |
| One pattern | 16 | -846.601 | 1788.743 |  | 1 |  |  |
| Two patterns | 25 | -397.954 | 945.189 | 865.865 | 0.982 | < .0001 | < .0001 |
| Three patterns | 34 | -113.081 | 429.186 | 321.305 | 0.978 | < .0001 | < .0001 |
| Four patterns | 43 | 154.392 | -52.02 | -188.457 | 0.983 | < .0001 | < .0001 |
| Five patterns | 52 | 349.329 | -388.152 | -553.146 | 0.988 | .0398 | < .0001 |
| Six patterns | 61 | 532.754 | -701.261 | -894.812 | 0.994 | .1239 | < .0001 |
| Seven patterns | 70 | 674.666 | -931.345 | -1153.452 | 0.995 | .2858 | < .0001 |
| Eight patterns | 79 | 813.942 | -1156.154 | -1406.819 | 0.977 | .4027 | < .0001 |
| *t*_2_: Context patterns after educational transition | df | LL | BIC | Sample-adjusted BIC | Entropy | VLMR | BLRT |
| One pattern | 16 | -771.067 | 1637.674 | 1586.907 | 1 |  |  |
| Two patterns | 25 | -220.888 | 591.058 | 511.734 | 0.992 | < .0001 | < .0001 |
| Three patterns | 34 | 174.046 | -145.069 | -252.950 | 0.992 | .0014 | < .0001 |
| Four patterns | 43 | 562.724 | -868.684 | -1005.122 | 0.995 | < .0001 | < .0001 |
| Five patterns | 52 | 797.91 | -1285.315 | -1450.309 | 0.997 | .0459 | < .0001 |
| Six patterns | 61 | 964.263 | -1564.28 | -1757.831 | 0.994 | .0840 | < .0001 |
| Seven patterns | 70 | 1106.292 | -1794.596 | -2016.703 | 0.994 | .1582 | < .0001 |
| Eight patterns | 79 | 1350.751 | -2229.772 | -2480.437 | 0.996 | .3472 | < .0001 |

*Fit indices for one to eight pattern solutions before and after educational transition* (*n* = 392)

*Note.* LL = model log likelihood; BIC = Bayesian Information Criterion; adjusted BIC = adjusted Bayesian Information Criterion; VLMR = Vuong-Long-Rubin likelihood ratio test; BLRT = bootstrap likelihood ratio test; LRT = likelihood ratio test.
