# Supplementary material for: Stability of context in sport and exercise across educational transitions in adolescence: hello work, goodbye sport club?
Source: BMC Public Health. 2022 Jan 21;22:152. doi: 10.1186/s12889-021-12471-4 (PMC8783455; doi:10.1186/s12889-021-12471-4)
Supplement: Supplementary file 3 — Additional file 3. [file 12889_2021_12471_MOESM3_ESM.docx]

*Table S3*

Associations between behavioural context patterns and types of sport and exercise (*t*_1_ and *t*_2_)

| Types of sport and exercise | Behavioural context patterns in sport and exercise | | | | | | | |
| --- | --- | --- | --- | --- | --- | --- | --- | --- |
|  | Non-club-organised sportspersons | | Mostly inactives | | Traditional competitive club athletes with friends | | Self-organised individualists | |
|  | *t*_1_ | *t*_2_ | *t*_1_ | *t*_2_ | *t*_1_ | *t*_2_ | *t*_1_ | *t*_2_ |
| Endurance sport (e.g. running) | 0.00% | 0.00% | 8.00% | 0.00% | 1.80% | 3.40% | 15.30% | 15.40% |
| Fitness (e.g. weight training) | 22.20% | 46.90% | 4.00% | 17.60% | 1.20% | 2.80% | 23.60% | 32.30% |
| Gymnastics | 0.00% | 2.00% | 0.00% | 0.00% | 3.60% | 1.40% | 9.70% | 0.00% |
| Athletics | 0.00% | 0.00% | 0.00% | 5.90% | 1.20% | 1.40% | 0.00% | 0.00% |
| Compositional-creative activities (e.g. dancing) | 44.40% | 26.50% | 0.00% | 5.90% | 11.5% | 15.20% | 9.70% | 7.70% |
| Release-oriented activities (e.g. yoga) | 0.00% | 2.00% | 24.00% | 0.00% | 0.00% | 0.70% | 0.00% | 3.10% |
| Outdoor- and mountain sports (e.g. skiing) | 4.40% | 2.00% | 0.00% | 11.80% | 2.40% | 1.40% | 9.70% | 10.80% |
| Sports games (e.g. football) | 15.60% | 14.30% | 48.00% | 47.10% | 68.50% | 66.90% | 27.80% | 18.50% |
| Martial arts (e.g. taekwondo) | 4.40% | 2.00% | 0.00% | 5.90% | 5.50% | 3.40% | 1.40% | 1.50% |
| Equestrian | 8.90% | 4.10% | 4.00% | 5.90% | 4.20% | 3.40% | 12.50% | 10.80% |
